# Identification of a novel heterozygous mutation in exon 50 of the *COL1A1* gene causing osteogenesis imperfecta

**DOI:** 10.1530/EDM-13-0002

**Published:** 2013-07-01

**Authors:** S A S Aftab, N Reddy, N L Owen, R Pollitt, A Harte, P G McTernan, G Tripathi, T M Barber

**Affiliations:** Division of Metabolic and Vascular Health, Clinical Sciences Research LaboratoriesWarwickshire Institute for the Study of Diabetes Endocrinology and Metabolism, University Hospitals Coventry and Warwickshire, University of WarwickClifford Bridge Road, Coventry, CV2 2DXUK; 1 Connective Tissue Disorders Service, Sheffield Diagnostic Genetics ServiceSheffield Children's NHS Foundation Trust, Western BankSheffield, S10 2THUK

## Abstract

A 19-year-old woman was diagnosed with osteogenesis imperfecta (OI). She had sustained numerous low-trauma fractures throughout her childhood, including a recent pelvic fracture (superior and inferior ramus) following a low-impact fall. She had the classical blue sclerae, and dual energy X-ray absorptiometry (DEXA) bone scanning confirmed low bone mass for her age in the lumbar spine (*Z*-score was −2.6). However, despite these classical clinical features, the diagnosis of OI had not been entertained throughout the whole of her childhood. Sequencing of her genomic DNA revealed that she was heterozygous for the c.3880_3883dup mutation in exon 50 of the *COL1A1* gene. This mutation is predicted to result in a frameshift at p.Thr1295, and truncating stop codon 3 amino acids downstream. To our knowledge, this mutation has not previously been reported in OI.

**Learning points:**

- OI is a rare but important genetic metabolic bone and connective tissue disorder that manifests a diverse clinical phenotype that includes recurrent low-impact fractures.
- Most mutations that underlie OI occur within exon 50 of the *COL1A1* gene (coding for protein constituents of type 1 pro-collagen).
- The diagnosis of OI is easily missed in its mild form. Early diagnosis is important, and there is a need for improved awareness of OI among health care professionals.
- OI is a diagnosis of exclusion, although the key diagnostic criterion is through genetic testing for mutations within the *COL1A1* gene.
- Effective management of OI should be instituted through a multidisciplinary team approach that includes a bone specialist (usually an endocrinologist or rheumatologist), a geneticist, an audiometrist and a genetic counsellor. Physiotherapy and orthopaedic surgery may also be required.

## Background

Osteogenesis imperfecta (OI) is a heterogeneous connective tissue disorder that manifests clinically as extreme bone fragility, brittleness and growth disorder (1). Although the commonest bone genetic disorder, OI is rare with a prevalence of 1/10 000 births (2). OI manifests a wide range of phenotypic characteristics and clinical severities with at least eight sub-types having been distinguished (2). The rarity, heterogeneity and variable severity of OI can make its effective diagnosis a clinical challenge. Early and effective management of OI through a multidisciplinary approach is important as delayed diagnosis of this condition can result in significant morbidity (avoidable through timely intervention) (1). In this case report, we present the case of a young woman with a new diagnosis of OI following multiple low-trauma fractures throughout her childhood, in whom we have identified a novel mutation in exon 50 of the *COL1A1* gene.

## Case presentation

The case is that of a 19-year-old female student who was referred to the Endocrine Clinic at the Warwickshire Institute for the Study of Diabetes, Endocrinology and Metabolism (WISDEM), University Hospitals Coventry and Warwickshire, following a low-impact fall that resulted in superior and inferior fractures of her pelvic rami. This injury was sustained when she tripped and fell onto the pavement from a standing position. Her medical history revealed numerous fractures, including tibia, clavicle and metacarpals, all resulting from low-impact falls. These fractures were sustained throughout her childhood, with the first one occurring at the age of 5 years. Other relevant history included current treatment for an eating disorder (anorexia nervosa). There was no family history of note, and in particular no family history of low-trauma fractures in her parents, brother or other family members. Her only medication was the combined oral contraceptive pill (Microgynon), which she had been taking for 3 years for contraceptive purposes. Prior to this, she had been oligo-amenorrhoeic (resulting from her being underweight). She was a smoker (10–15 cigarettes/day) and only rarely drank alcohol. On systemic enquiry, she had a history of recurrent chest infections and poor hearing. She was born at term (normal vaginal delivery) and had normal early development, having achieved early-life developmental milestones (including physical, emotional, language, social and cognitive development) at appropriate ages.

On examination, she was underweight with a BMI of 17.9 kg/m^2^. Her weight was 52 kg and height was 170.3 cm. She had blue discolouration to her sclerae bilaterally, but her dentition was good. Other than bilateral clinodactyly, her musculoskeletal examination revealed no deformity, tenderness or weakness, and there was no chest wall deformity. It was, however, noted that she had some increased laxity to the joints in her upper limbs (including elbow, meta-carpal and phalangeal joints in both arms and hands). While Rinne's test was positive on both sides, Weber's test localised to the right side, suggesting either partial ipsilateral conductive hearing defect or (more likely) contralateral sensorineural hearing loss.

## Investigation

**Figure 1:**
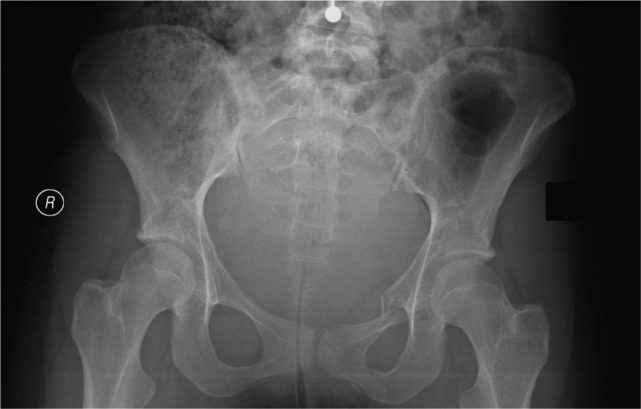
X-ray of pelvis demonstrating fractures of the left superior and inferior pubic rami.

Biochemically, her full blood count and renal and liver functions were normal, and her serum calcium, magnesium, phosphate, alkaline phosphatase and 25-hydroxycholecalciferol levels were within their normal ranges. Baseline P1NP was in the lower part of the normal range (due to the reduced production of type 1 collagen in OI type 1). Her pelvic X-rays confirmed fractures to the superior and inferior rami (Fig. 1). The dual energy X-ray absorptiometry (DEXA) bone scan revealed low bone mass for her age within her lumbar vertebrae (*Z*-score −2.6 (low bone mass for age being defined as a *Z*-score <−1.0)) but normal bone mass for her age in her hips (*Z*-score −0.8 (normal bone mass for age being defined as a *Z*-score >−1.0)). Sequencing of her genomic DNA revealed that she is heterozygous for the c.3880_3883dup mutation in exon 50 of the *COL1A1* gene. This mutation is predicted to result in a frameshift at p.Thr1295 and truncating stop codon 3 amino acids downstream.

## Treatment

Following confirmation of a diagnosis of OI type 1, the initial step in her management was to advise her on lifestyle modifications, particularly in relation to her occupation. She worked part-time in a supermarket, and she was advised to avoid any lifting of heavy loads. Furthermore, with the patient's consent, a letter was written to her employer outlining her new diagnosis and the importance of avoiding any heavy lifting or other physical activities that may put her at risk of further fracture. Prior to initiation onto bisphosphonate therapy, she was fully informed and counselled regarding the potential teratogenic effects of these agents and to avoid pregnancy while on this drug. Following her consent, weekly alendronate therapy was commenced on her with calcium and vitamin D supplementation. Her response to bisphosphonate therapy will be monitored at future appointments with repeat DEXA bone scans. She will continue with the combined oral contraceptive pill. She has no plans for pregnancies but this will be discussed at future appointments and a decision regarding future bisphosphonate therapy made accordingly. She was referred to a geneticist and genetic counselling for further input in relation to the implications of her newly identified novel mutation for her, any future offspring and her relatives. She was advised regarding pregnancy and the need for close multidisciplinary follow-up during any future pregnancy and the possible complications for both her and the foetus during any future pregnancy. In view of her hearing impairment, she was referred to audiology for formal assessment of her hearing and further follow-up.

## Outcome and follow-up

The patient made excellent progress and has follow-up in a multidisciplinary outpatient setting. She has had no further fractures since implementation of her lifestyle advice. She has had genetic counselling and has excellent understanding of her condition and the implications for any future pregnancy. She tolerates bisphosphonate therapy and has regular audiometry assessments. Management is currently successful through lifestyle, conservative and pharmacological-based therapies. We plan to continue the alendronate therapy for a period of 3 years initially to assess for any beneficial effects on bone mineral density. Owing to the incorporation of bisphosphonates into the bone matrix and subsequent release back into the serum, there is a theoretical teratogenic risk of these agents even following their discontinuation. Therefore, avoidance of pregnancy and use of a reliable form of contraception is important during therapy with a bisphosphonate and for at least 1 year following its discontinuation.

## Discussion

OI is a heterogeneous connective tissue disorder that results from defective production of type 1 collagen. Although OI is the commonest genetic bone disorder, its overall prevalence is rare and estimated to be 1 in 10 000–20 000 births (2). Characterised by bone fragility, OI manifests clinically as increased susceptibility to bone fractures (3) that often result from low-impact trauma (4) (as illustrated in this case). Although screening for OI should be considered in patients sustaining unusual low-impact fractures, it should also be noted that pelvic fracture is very unusual even in patients with OI of this age. It is noteworthy in this case that she had a co-existing diagnosis of anorexia nervosa, which is a major risk factor for low bone mass and fractures. This is relevant to her presentation in late adolescence and is likely to have been a contributory factor (augmenting the effects of OI) to her history of recurrent low-trauma fractures.

The phenotype of OI is diverse and is influenced by the genetic mutation responsible. This combined with the rarity of OI presents a diagnostic challenge (5). The case described here has the mildest form of OI type 1, which is also the most commonly encountered subtype (1) (3) and is associated with a propensity towards low-trauma fractures (5) (6). A common manifestation of OI type 1 (also demonstrated in this case) is presenile sensorineural hearing loss (5). There are in excess of 800 mutations associated with OI including more than ten reported mutations occurring within exon 50 of the *COL1A1* gene (7) (8) (9) (10). Patients with OI manifest heterozygous mutations (presumably because homozygous variants die *in utero*). The vast majority of patients with OI (90%) have a point mutation in one of two genes: *COL1A1* or *COL1A2*. These genes code for protein constituents of type 1 pro-collagen (proa1 and proa2 chains respectively) (3). OI type 1 results from mutations in *COL1A1* that cause a quantitative defect of type 1 collagen. Genome sequencing in this case identified heterozygosity for the c.3880_3883dup mutation in exon 50 of the *COL1A1* gene. This mutation produces a frameshift at p.Thr1295 that results in a truncating stop codon 3 amino acids downstream. Although there are several similar mutations published in this region of *COL1A1* (some of which have similar phenotypes; Table 1 (7) (8) (9) (10)), to our knowledge, the mutation in *COL1A1* identified here has not previously been reported in OI.

**Table 1:** Outline of some reported mutations in exon 50 of the *COL1A1* gene associated with OI. Data are also shown for type of mutation, affected protein, downstream effect and sub-type of OI reported. Details of the novel mutation discussed in this case report are also included

**Exon location**	**Reported change in DNA**	**Initial effect of the mutation**	**Effect on downstream protein**	**Further effects**	**OI type** (clinical/phenotype)	**Reference**
50	c.3829 G→C	Substitution Missense	p.Asp1277His	Asp1099His	I, II	(7)
50	c.3831 C→G	Substitution Missense	p.Asp1277Glu	–	II	(8)
50	c.3880_3883dup	Duplication Frameshift	p.Thr1295	Truncating stop codon 3 amino acids downstream	I	Our case
50	c.3897 C→G	Substitution Missense	p.Cys1299Trp	Cys1121Trp	I	(9)
50	c.3910 C→T	Substitution Nonsense	p.(Gln1304*)	Gln1162Stop	I	(10)

The challenge of diagnosing OI in clinical practice is heightened when there is a lack of family history for the condition or its characteristic features. While bilateral blue sclerae are a phenotypic characteristic of OI, the differential diagnosis of this clinical sign includes numerous other conditions including Ehlers–Danlos syndrome and Marfan's syndrome. OI is a diagnosis of exclusion, and there are no minimum diagnostic criteria. The key diagnostic assessment for OI is genetic testing of blood or saliva specimens for *COL1A1* mutations (6) (dermal fibroblast culture from skin biopsies (to identify pro-collagen expression) can also be useful diagnostically (2) (3)). Genotyping for suspected cases of OI is an expensive investigation and should be considered carefully in each suspected case. Genetic testing is often indicated when there is clinical suspicion of OI associated with some atypical features. However, the presence of clear clinical features of OI limits the utility of genotyping and often renders this investigation unnecessary. Furthermore, the presence of clear clinical features of OI often enables effective management to be implemented, and clinical genetics advice is provided (including advice about future offspring) solely based on phenotype.

Despite the classical clinical presentation of OI, and the frequent assessments by numerous health care professionals following recurrent low-impact fractures in this case, it is interesting that a diagnosis of OI was not entertained earlier. The rarity of OI and the apparent lack of awareness of this condition among health care professionals may have been contributory. Improving awareness of OI, particularly among ‘front-line’ NHS staff (including GPs and emergency care doctors), should be a priority.

Management of OI should be through a multidisciplinary approach that includes input from a specialist in bone health (endocrinologist or rheumatologist), audiologist, geneticist, genetic counsellor and occasionally physiotherapist and orthopaedic surgeon. The potential effects of any future pregnancy on the mother and foetus need to be considered carefully. The principals of effective management of OI are to reduce the risk of fractures, minimise skeletal deformity and maximise quality of life (2). Management of OI type 1 typically comprises a combined conservative and pharmacological approach: avoidance of heavy lifting and impact sports; use of splints; physiotherapy to address joint laxity; and vitamin D and calcium supplements. Regular (multidisciplinary) outpatient follow-up is required. With regard to bisphosphonate therapy in OI, there is little evidence in the literature that these agents actually improve fracture rates, although they have been shown to improve bone mineral density. The optimal duration of bisphosphonate therapy in OI is not clear, and future studies are required to address this. The use of bisphosphonate therapy in women of childbearing age should be considered carefully. Each patient should be counselled appropriately prior to the initiation of any bisphosphonate therapy regarding the potential teratogenic effects of these agents (both during their use and following their discontinuation). Clearly, a reliable form of contraceptive should be used while on these agents and for at least a year following their discontinuation.

To conclude, we present the case of a young woman with a long-standing history of low-impact fractures and the characteristic bilateral blue sclerae of OI type 1. Genetic testing revealed a heterozygous novel frameshift mutation (c.3880_3883dup) in exon 50 of the gene *COL1A1* that results in a truncating stop codon. This case illustrates the importance of early diagnosis (and potential for delayed diagnosis) and instigation of an effective management plan through a multidisciplinary approach in patients with OI. To our knowledge, this is also the first report of the novel mutation, c.3880_3883dup within exon 50 of the *COL1A1* gene presenting with the phenotype of OI type 1.

## Patient consent

Informed consent has been obtained from the patient for publication of this (anonymised) submitted case report and accompanying images.

## Author contribution statement

All authors listed contributed substantially to the preparation of this manuscript. The corresponding author (T M Barber) is the named physician of the patient.
